# Bioinformatic analysis, expression analysis, and subcellular localization of GeBP transcriptional regulator family in response to abiotic stress in *Brassica napus*

**DOI:** 10.3389/fpls.2026.1858194

**Published:** 2026-06-17

**Authors:** Sana Basharat, Wajid Saeed, Pingwu Liu, Muhammad Waseem

**Affiliations:** 1 School of Breeding and Multiplication (Sanya Institute of Breeding and Multiplication), School of Tropical Agriculture and Forestry, Hainan University, Sanya, China; 2 Fang Zhiyuan Academician Team Innovation Center, Hainan, Haikou, China; 3 Key Laboratory for Quality Regulation of Tropical Horticultural Crops of Hainan Province, Hainan, Haikou, China

**Keywords:** abiotic stress, *BnaGeBP*, *Brassica napus*, phylogeny, spatiotemporal expression

## Abstract

**Introduction:**

The GLABROUS1 enhancer-binding protein (GeBP) gene family represents a plant-specific class of transcriptional regulators involved in plant growth, development, and adaptation to environmental stresses. Although GeBP proteins have been characterized in *Arabidopsis*, systematic analysis of GeBP genes in *Brassica napus* and their responses to abiotic stresses remains limited.

**Methods:**

In this study, we performed a genome-wide identification and characterization of GeBP genes in B. napus. Chromosomal distribution, phylogenetic relationships, gene duplication events, exonintron organization, conserved domains, promoter cis-regulatory elements, tissue-specific expression patterns, subcellular localization, and stress-responsive expression profiles under salt, drought, heat, and cold stresses were analyzed.

**Results:**

A total of 35 BnaGeBP proteins were identified and found to be unevenly distributed across the B. napus chromosomes. Phylogenetic analysis classified the BnaGeBP proteins into three distinct groups together with GeBP homologs from selected monocot and dicot species. Duplication analysis indicated that the expansion of the BnaGeBP family was mainly driven by segmental duplication events, with 20 segmental duplication pairs and one tandem duplication pair identified. Gene structure and conserved domain analyses supported the phylogenetic conservation of BnaGeBP genes. Promoter analysis revealed diverse hormone- and stress-responsive cis-regulatory elements, including drought- and low-temperature-responsive motifs. Tissue expression profiling showed variable spatial expression patterns, while selected BnaGeBP genes displayed differential expression under salt, drought (PEG6000), heat, and cold stress treatments.

**Discussion:**

This comprehensive analysis provides valuable insights into the evolution, structural conservation, and potential functions of the BnaGeBP gene family in *B. napus*. The stress-responsive expression patterns suggest that selected BnaGeBP genes may play important roles in abiotic stress adaptation, providing a foundation for future functional studies and genetic improvement of stress tolerance in *B. napus*.

## Introduction

Unfavorable climate changes threaten plant growth and agricultural productivity, posing a challenge to global food security. Plants have evolved intricate transcriptional and posttranscriptional processes to withstand these climatic changes (Movahedi et al., 2024). Transcription factors (TFs) are reported to play a critical role in abiotic stress tolerance by triggering a range of defense systems (Bhoite et al., 2025). TFs function by activating or suppressing gene expression or transcription, often through specific binding to nucleotide sequences within promoter regions (Waseem et al., 2022). In plants, a total of 58 families of TFs regulates thousands of genes within regulatory networks, enhancing plant growth, development, and response to diverse biological processes (Wai et al., 2022). Some of these TF include *AP2/ERF (APETALA2/*ethylene-responsive element binding factor), *ATAF* (Arabidopsis transcription activation factor), bHLH (basic Helix Loop Helix), bZIP (basic leucine zipper), MYB (myeloblastosis), NAC (NAM-no apical meristem), CUC (cup-shaped cotyledon), and WRKY (Waseem et al., 2022). The *GLABROUS* enhancer binding protein (GeBP) is a plant-specific TF initially identified in Arabidopsis in 2003 (Curaba et al., 2003). GeBP TFs contain a central DNA-binding domain and a C-terminal putative leucine-zipper pattern (Wu et al., 2024).

Several studies have reported the role of GeBP proteins in plant development. For instance, GeBP proteins are critical for cell expansion (Perazza et al., 2011) and trichome formation (Curaba et al., 2003). In Arabidopsis, a nonconical GeBP interacts with *AtGL1* to regulate trichome formation (Chevalier et al., 2007). In tea plants, Zhou et al. (2023) demonstrated that VIGS-mediated silencing of *CsGeBP4* resulted in a high-density trichome phenotype, revealing its role in trichome formation. In apple, overexpression of *GeBP3* resulted in reduced ABA sensitivity, promoting adventitious root formation and delaying flowering (Liu et al., 2023). Additionally, several studies have highlighted the complex role of GeBP proteins in stress tolerance. In Arabidopsis, *AtGPL4* functions as a negative regulator of root growth and responds rapidly to heavy metal exposure, including cadmium, copper, and zinc (Khare et al., 2017). In *Brassica rapa*, the genes *BrGeBP3, BrGeBP14*, and *BrGeBP17* show significantly increased expression under drought stress. Notably, *BrGeBP3* and *BrGeBP14*—homologs of the Arabidopsis gene *At5g28040* —interact with DES1 to promote hydrogen sulfide (H_2_S) biosynthesis. This H_2_S production modulates *SnRK2.6*-mediated ABA signaling, leading to enhanced stomatal closure and improved drought tolerance (Wang et al., 2023). In contrast, overexpression of *MdGeBP3* in Arabidopsis resulted in increased sensitivity to drought, an effect attributed to accelerated chlorophyll degradation and excessive accumulation of reactive oxygen species (H_2_O_2_ and O_2_^-^). This oxidative damage disrupted membrane stability, establishing *MdGeBP3* as a negative regulator of drought resistance (Liu et al., 2023).

The identification of GeBP TF gene family has been performed in several plant species including Arabidopsis (23 members) (Chevalier et al., 2007), apple (7 members) (Liu et al., 2023), *Brassica rapa* (20 members) (Wang et al., 2023), soybean (9 members), rice (13 members), tea (6 members), tomatoes (11 members) (Cao et al., 2025). Increased identification of GeBP TF genes has facilitated a deeper understanding of their biological roles. However, the evolution and functional divergence of GeBP genes in *Brassica napus* remained unclear. *B. napus*, a key member of the Brassicaceae family, holds the second position in global oilseed cultivation for edible oil production (Ilyas et al., 2024) and is sensitive to environmental constraints that impact canola growth and reduce oil quantity and quality (Ali et al., 2023). GeBP TFs perform a variety of important biological functions and play a critical regulatory role in plant growth, development, and stress resistance. Therefore, identifying and elucidating the biological functions of the GeBP gene family in *B. napus* is of significant importance.

*B. napus* is one of the important oilseed crops and sensitive to adverse environmental conditions such as salinity, drought, and temperature extremes, which severely impact overall plant growth, seed yield, and oil quality. Understanding stress-responsive transcription factor families in *B. napus* is therefore essential for elucidating molecular adaptation mechanisms and developing stress-resilient cultivars. In particular, transcriptional regulators involved in abiotic stress signaling are considered important targets for future crop improvement programs. Despite the availability of the *B. napus* reference genome, no systematic genome-wide investigation of the GeBP transcriptional regulator family has previously been reported in this species. Therefore, a comprehensive analysis of *BnaGeBP* genes, including their phylogenetic relationships, conserved motifs, gene duplication patterns, promoter *cis*-regulatory elements, expression profiles under abiotic stresses, and subcellular localization, is necessary to better understand their potential biological functions and regulatory roles in stress adaptation. In this study, a comprehensive genome-wide investigation of the GeBP TF family members was conducted using the *B. napus* (ZS11) genome. We further analyzed intron-exon distribution, conserved domains and motifs, chromosomal locations, phylogeny, and gene duplication via synteny analyses. Additionally, we examined spatial and temporal expression patterns in different plant parts under abiotic stress, including salinity, drought (using PEG 6000), and temperature extremes (cold, 4 °C, and heat, 30 °C). These results will contribute to a greater understanding of the evolution and biological functions of *B. napus* GeBP genes.

## Materials and methods

### Mining of *B. napus* GeBP proteins

To identify the GeBP gene family in the *B. napus* proteome, we employed two approaches. First, the Arabidopsis GeBP protein, retrieved from the TAIR genome (https://www.arabidopsis.org/), was used as a query in the *B. napus* proteome via BLASTP (E-value: 1 × 10^− 5^). Secondly, the DUF domain, (DUF573, PF04504) pfam pattern was obtained from the Pfam database (https://www.ebi.ac.uk/interpro/entry/pfam/#table) and hmmsearch (with E-value: 1 × 10^−5^) algorithm was used to identify putative GeBP proteins in *B. napus* proteome. Furthermore, the NCBI conserved domain database (NCBI CDD, https://www.ncbi.nlm.nih.gov/Structure/bwrpsb/bwrpsb.cgi) and Simple Modular Architecture Research Tool (SMART) (http://smart.embl.de/smart/change_mode.cgi) were utilized to validate the presence of the DUF573 domain in each deduced sequence. Finally, incomplete, redundant, or sequences lacking the complete domain were removed to produce a final set of validated GeBP family genes. The GeBP proteins were designated as *BnaGeBP* (utilized subsequently in analysis) and numbered sequentially on the chromosomes. Additionally, to assess the physicochemical characteristics of the BnaGeBP proteins–including peptide length, grand average of hydropathy (GRAVY), molecular weight (Da), isoelectric point (pI), and instability index–these parameters were calculated using the Sequence Manipulation Suite (https://www.bioinformatics.org/sms2/). Finally, *in silico* subcellular localization predictions were performed for each BnaGeBP protein using WoLF–PSORT (https://wolfpsort.hgc.jp/), and these predictions were further validated with BUSCA (http://busca.biocomp.unibo.it/).

### Phylogeny of *B. napus BnaGeBP* genes

To elucidate the phylogenetic relationship and potential function of *B. napus* GeBP protein, the peptide sequences of *BnaGeBP* from Arabidopsis (23 members) (Chevalier et al., 2007), *B. rapa* (20 members) (Wang et al., 2023), soybean (9 members), rice (13 members), tomatoes (11 members) (Cao et al., 2025) and maize were retrieved from phytozome database (https://phytozome.jgi.doe.gov/pz/portal.html). A comprehensive phylogenetic tree was constructed using the IQTREE program (http://iqtree.cibiv.univie.ac.at/) employing the maximum likelihood (ML) algorithm with Poisson substitution model, pairwise deletion of gaps, and bootstrap set at 1000 replicates.

### Structural characterization of *BnaGeBP*

The position of the *BnaGeBP* domain in peptide sequences was predicted using NCBI CDD. The intron-exon distribution of all *B. napus* GeBP genes was retrieved from the *B. napus* genome annotation. To explore conserved motif sequences within the BnaGeBP protein, the MEME suite (https://meme-suite.org/meme/tools/meme) was used with the following parameters: (*i*) a maximum number of motifs was set to 10, (*ii*) any number of repetitions, and (*iii*) an optimum motif width of ≥ 10 and ≤ 50. The output from the MEME tools was visualized using tbtools (Chen et al., 2023).

### *BnaGeBP* gene chromosomal location and duplication analysis in *B. napus* genome

Chromosome sizes and chromosomal locations for *BnaGeBP* genes were obtained from *B. napus* genome annotation, and precise chromosomal locations were determined using Tbtools. Gene duplication events, including segmental and tandem duplications, were analyzed using MCScanX (https://github.com/wyp1125/MCScanX). A syntenic map illustrating chromosome locations and duplications was visualized using a circos plot in Tbtools. The Ka/Ks ratios of each *BnaGeBP* paralogous gene pair were calculated using the Ks_Ka calculator (https://sourceforge.net/projects/kakscalculator2/). Divergence time (T, millions of years ago) was calculated as T = Ks/2R, where R = 1.5 × 10−8 substitutions per site per year (Shah et al., 2024).

### Plant materials, growth, and stress treatment

The plant material used in this study was *B. napus* 383-5, which is an inbred line developed by our group at the School of Breeding and Multiplication (Sanya Institute of Breeding and Multiplication), School of Tropical Agriculture and Forestry, Hainan University, Sanya, China, and preserved in our laboratory (Basharat et al., 2025). The seeds were surface sterilized and grown in a growth room under controlled conditions, maintained at 24 °C with a 16/8 h light/dark photoperiod. To evaluate the expression of *BnaGeBP* genes, tissue samples including stem, root, leaf, flower, seed, and siliques were collected. To evaluate abiotic stress responses, two-week-old B. napus seedlings were treated with 150 mM NaCl, 10% PEG6000 for drought simulation, heat (30 °C), and cold (4 °C). Plants treated with water served as controls. Samples were collected at 0 h, 3 h, 6 h, 12 h, and 24 h after treatment. In addition, we clarified the control treatment description by stating that samples collected at 0 h prior to stress exposure were used as the control (CK) for comparison with all subsequent stress-treatment time points. All harvested samples were immediately snap-frozen in liquid nitrogen and stored at −80 °C for later analysis. Each collected sample contained five biological replicates, each with three technical replicates.

### Transient expression analysis of *BnaGeBP* in tobacco leaves

Full-length sequences of *BnaA.GeBP4, BnaA.GeBP16*, and *BnaC.GeBP20*, lacking stop codons, were amplified and cloned into the pGreen0029-GFP vector. Recombinant plasmids containing the *BnaA.GeBP4::GFP, BnaA.GeBP16::GFP*, and *BnaC.GeBP20::GFP* fusion genes were generated. A plasmid expressing empty pGreen0029-GFP with backbone expressing GFP alone without insertion of any *BnaGeBP* coding sequence served as a control. These recombinant plasmids were transformed into tobacco leaf epidermal cells via *Agrobacterium*-mediated transformation. Fluorescence was observed in the tobacco leaves using a confocal laser scanning microscope (Olympus FV10I, Leica, Germany), and photographs were acquired.

### Nucleic acid extraction and RT-qPCR analysis

Total RNA was extracted from all collected samples using TRIZOL reagent, following the manufacturer’s instructions. The extracted RNA was quantified using a Nanodrop (Thermo Fisher Scientific, USA), and its quality was assessed via 2% (w/v) agarose gel electrophoresis. To verify the expression of *BnaGeBP* genes in *B. napus*, fifteen *BnaGeBP* genes were randomly selected from the identified gene family members for RT-qPCR expression analysis under abiotic stress conditions. The selected genes represented different phylogenetic groups and chromosomal distributions within the *BnaGeBP* family. The cDNA was synthesized using Prime Script ™ RT reagent Kit with gDNA Eraser (Takara, JAPAN). RT-qPCR was performed using SYBR-Premix Ex Taq-II (TliRNaseH Plus) according to the supplied protocol, and reactions were run on a CFX96 Touch ™ Real-Time PCR Detection System (BIO-RAD, USA). *BnACTIN7* was used as an internal reference gene. Relative expression levels of each *BnaGeBP* were assessed using the 2^−ΔΔCt^ method (Livak and Schmittgen, 2001). The primers used in this study are listed in **Supplementary Table 1**.

### Statistical analysis

Experiments were conducted with three biological replicates, each comprising three technical replicates. Gene expression differences between treatments and controls were assessed using Student’s t-test, with statistical significance defined at *P* < 0.05. Error bars represent standard deviation.

## Results

### Mining of GeBP genes in *B. napus*

We performed BLAST and HMMER searches to identify GeBP TF gene family members in *B. napus*, followed by further sequence validation by NCBI CDD and SMART database. A comprehensive screening identified 35 members of the GeBP gene family within the *B. napus* whole proteome. All identified GeBP genes were named according to their chromosomal location, in ascending order: *BnaA.GeBP1* to *BnaA.GeBP16* for genes located on subgenome A, and *BnaC.GeBP17* to *BnaC.GeBP35* for genes located on subgenome C. The GeBP proteins vary in sequence length, pI, and molecular weight. Peptide lengths ranged from 252 amino acid residues with a molecular weight of 28.38 kDa (*BnaA.GeBP7)* to 624 amino acids with a molecular weight of 71.09 kDa (*BnaC.GeBP17)*. The pI values ranged from 4.45 (*BnaA.GeBP9*) to 9.74 (*BnaC.GeBP30*), and analysis of the GRAVY score indicated that all proteins were hydrophilic. *In-silico* subcellular localization analysis suggested that the majority of *BnaGeBPs* were localized in the nucleus, with exceptions observed for *BnaA.GeBP1* and *BnaC.GeBP19*, which were located in the cytoplasm, and *BnaC.GeBP17*, which was localized in the chloroplast. Details of the *BnaGeBP* family members in *B. napus* are presented in **Table 1**.

**Table 1:** Features of GeBP gene family members in *B. napus*.

Gene ID	Gene name	aa	MW	pI	GRAVY	*In-silico* subcellular localization	Chromosome	Start	End	Strand
BnaA01T0144400ZS	*BnaA.GeBP1*	265	29.86617	5.77	-0.456	cyto	A01	8580314	8583398	+
BnaA01T0147500ZS	*BnaA.GeBP2*	352	38.7246	6.33	-0.798	nucl	A01	8729873	8730931	+
BnaA01T0400700ZS	*BnaA.GeBP3*	403	44.89415	5.95	-0.74	nucl	A01	35730746	35732236	+
BnaA03T0484500ZS	*BnaA.GeBP4*	365	40.43847	5.72	-0.935	nucl	A03	26824403	26825500	+
BnaA03T0581900ZS	*BnaA.GeBP5*	412	45.72543	5.24	-1.009	nucl	A03	41997236	41998474	+
BnaA03T0584200ZS	*BnaA.GeBP6*	308	34.67391	8.88	-1.012	nucl	A03	42584654	42585580	–
BnaA03T0585600ZS	*BnaA.GeBP7*	252	28.38017	9.37	-0.972	nucl	A03	42772616	42773446	–
BnaA04T0130400ZS	*BnaA.GeBP8*	304	34.25249	5.37	-0.931	nucl	A04	14671971	14672885	+
BnaA04T0233800ZS	*BnaA.GeBP9*	391	44.62934	4.45	-0.938	nucl	A04	22000474	22002660	+
BnaA05T0143400ZS	*BnaA.GeBP10*	302	34.31745	5.42	-0.845	nucl	A05	8879178	8880325	+
BnaA05T0479600ZS	*BnaA.GeBP11*	425	46.79599	4.84	-0.638	nucl	A05	43458703	43459980	–
BnaA06T0074500ZS	*BnaA.GeBP12*	356	39.0964	8.23	-0.936	nucl	A06	4353757	4354827	–
BnaA06T0352400ZS	*BnaA.GeBP13*	616	66.82404	4.97	-0.385	nucl	A06	42769365	42772099	–
BnaA07T0085100ZS	*BnaA.GeBP14*	284	31.71084	8.25	-0.663	nucl	A07	13590262	13592150	+
BnaA07T0175100ZS	*BnaA.GeBP15*	461	53.26358	9.21	-0.858	nucl	A07	20007293	20009658	–
BnaA09T0159700ZS	*BnaA.GeBP16*	380	42.03269	5.15	-1.015	nucl	A09	10156628	10157987	+
BnaC01T0183400ZS	*BnaC.GeBP17*	624	71.09959	9.04	-0.34	chlo	C01	13544752	13551442	+
BnaC01T0187500ZS	*BnaC.GeBP18*	364	40.03	6.88	-0.894	nucl	C01	13873967	13875061	+
BnaC01T0514000ZS	*BnaC.GeBP19*	350	39.12085	5.74	-0.703	cyto	C01	57673999	57675110	–
BnaC04T0237800ZS	*BnaC.GeBP20*	368	40.6534	5.58	-0.898	nucl	C04	32747387	32748493	–
BnaC04T0361200ZS	*BnaC.GeBP21*	368	40.27339	5.95	-0.73	nucl	C04	48225962	48227068	–
BnaC04T0374500ZS	*BnaC.GeBP22*	315	35.75212	7.62	-1.063	nucl	C04	49822735	49823682	–
BnaC04T0374600ZS	*BnaC.GeBP23*	287	32.39837	8.59	-1.078	nucl	C04	49827297	49828656	–
BnaC04T0374900ZS	*BnaC.GeBP24*	310	35.42603	8.97	-0.985	nucl	C04	49846932	49847864	–
BnaC04T0419000ZS	*BnaC.GeBP25*	308	34.80702	5.81	-1.023	nucl	C04	54445316	54446242	+
BnaC04T0549900ZS	*BnaC.GeBP26*	450	51.18651	4.47	-0.949	nucl	C04	66722699	66725121	+
BnaC05T0092100ZS	*BnaC.GeBP27*	399	43.54114	6.9	-0.944	nucl	C05	5252373	5253572	–
BnaC05T0542200ZS	*BnaC.GeBP28*	425	46.78005	4.88	-0.64	nucl	C05	56293128	56294405	–
BnaC06T0153300ZS	*BnaC.GeBP29*	301	34.15813	5.02	-0.826	nucl	C06	24841783	24842980	–
BnaC06T0168600ZS	*BnaC.GeBP30*	304	34.41829	9.77	-0.838	nucl	C06	26993847	26996337	–
BnaC07T0131000ZS	*BnaC.GeBP31*	308	35.20769	9.29	-0.942	nucl	C07	25263945	25265671	+
BnaC07T0341300ZS	*BnaC.GeBP32*	411	44.85504	4.87	-0.581	nucl	C07	47553284	47554929	+
BnaC07T0462600ZS	*BnaC.GeBP33*	370	40.86202	5.95	-0.901	nucl	C07	55891606	55892718	+
BnaC08T0071700ZS	*BnaC.GeBP34*	357	40.31389	6.96	-0.811	nucl	C08	9384767	9385869	–
BnaC09T0176000ZS	*BnaC.GeBP35*	379	42.04676	5.2	-1.043	nucl	C09	14549234	14550373	+

aa, Number of Amino Acid; Molecular Weight, MW (Kda); Instability index, pI; Grand Average of Hydropathicity, GRAVY.

### Phylogenetic analyses and classification of the *B. napus* GeBP gene family

**Figure 1:**
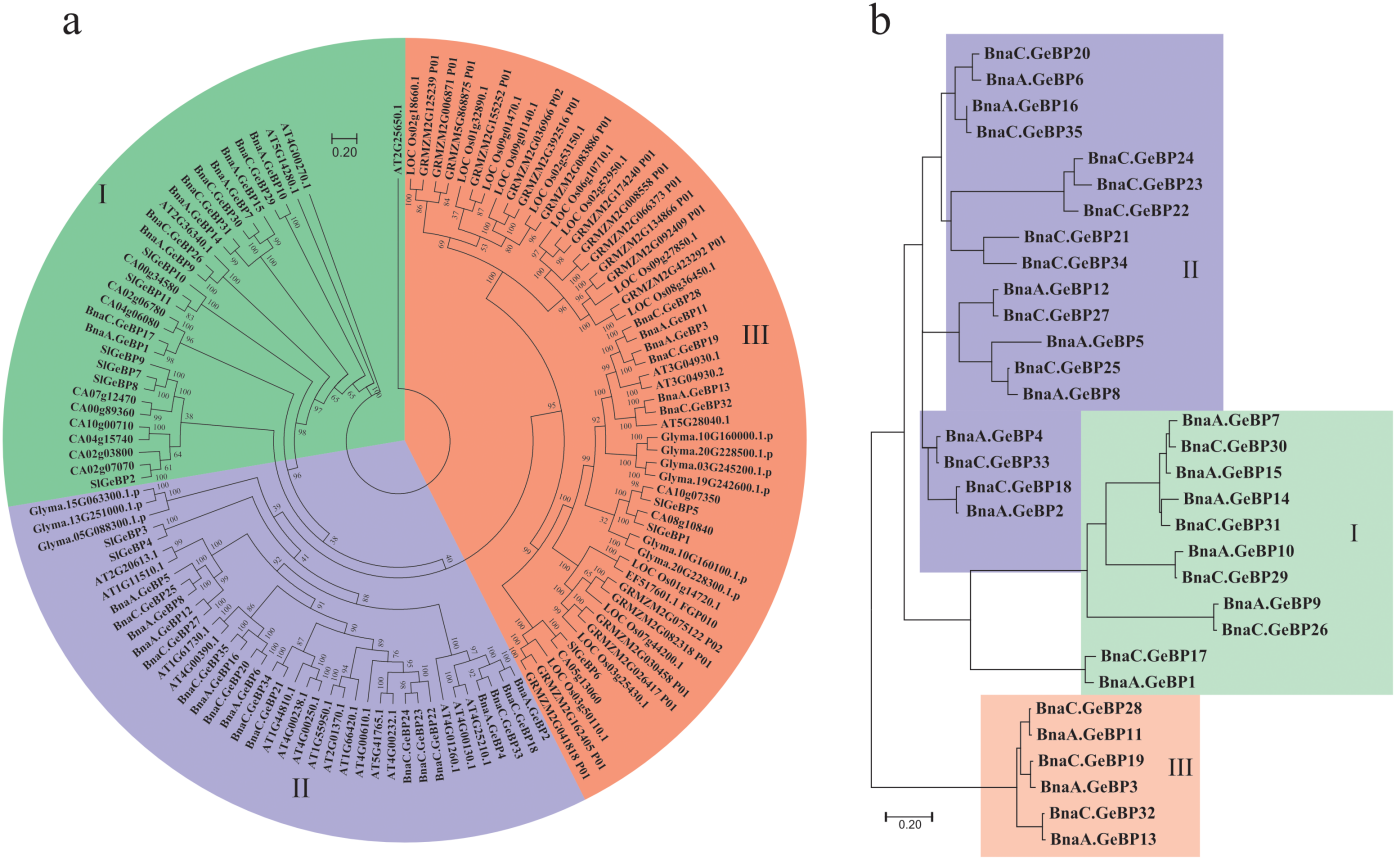
An unrooted maximum likelihood (ML) tree of BnaGeBP proteins **(a)** a maximum likelihood (ML) phylogeny of BnaGeBP proteins from *B. napus*, Arabidopsis, rice, and soybean **(b)** Phylogeny of BnaGeBP proteins only with bootstrap of 1000 replicates were generated in IQ-Tree program. All *BnaGeBP* members were clustered into three clades and are represented by different colors. Protein designations are as follows: Bna = *Brassica napus*, AT = Arabidopsis, GRMZM = soybean, Sl = tomato, CA = pepper, and LOC = rice.

A phylogenetic tree was constructed using peptide sequences from various plant species such as, Arabidopsis, tomato, rice, soybean, pepper, and maize to predict the possible biological function and evolutionary role of *BnaGeBP* genes in *B. napus*. The resulting phylogeny grouped all GeBP genes into three clusters: I, II, and III (**Figure 1a**). Cluster III contained the largest number of members (54), followed by Cluster II with 39 and Cluster I with 30 members (**Figure 1b**). Notably, Clusters I and II were exclusively composed of GeBP members from dicotyledonous plants, comprising nine and twenty GeBP members, respectively. Cluster III consisted of six GeBP members from *B. napus*, alongside 48 members from rice, maize, pepper, soybean, Arabidopsis, and tomato, suggesting a closer evolutionary relationship (**Figure 1**).

### Sequence analysis of *B. napus* GeBP family

**Figure 2:**
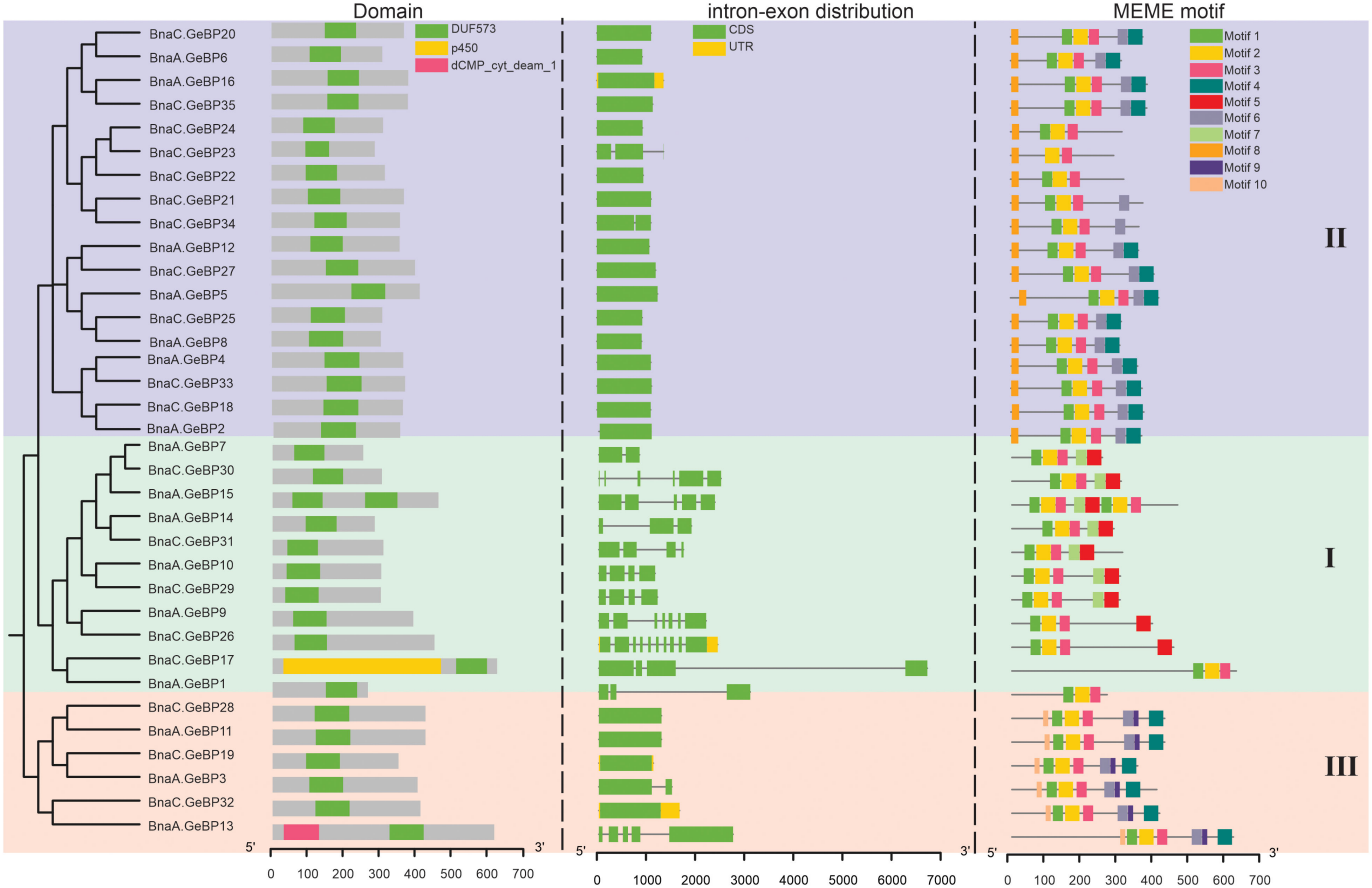
An analysis of the *BnaGeBP* gene, including the identification of the DUF573 domain, intron-exon distribution, and conserved motifs. This analysis was performed using the MEME suite, targeting ten motifs per gene. Sequence lengths are indicated by gray lines, with blocks of different colors representing conserved motifs. Black lines denote introns, yellow boxes represent exons, and green boxes represent untranslated regions (UTR).

The functional characteristics of *BnaGeBP*, a protein domain, were investigated alongside its intron-exon distribution and organization of conserved motifs using the MEME suite and phylogenetic analysis (**Figure 2**). Conserved domain analysis revealed that *BnaGeBP* possesses a characteristic domain present in all putative sequences, with additional domains observed in *BnaC.GeBP17* and *BnaA.GeBP13*. Regarding intron-exon distribution, genes within groups II and III exhibited single introns, with the exception of *BnaC.GeBP23/BnaA.GeBP34* in group II and *BnaA.GeBP3/BnaA.GeBP13* in group III. *BnaGeBP* genes in group I demonstrated gain and loss of introns, containing a varying number (2 – 10) (**Figure 2**).

We further explored the presence of conserved motifs in *BnaGeBP* genes, identifying ten conserved motifs (**Figure 2**). Notably, the composition of these motifs was similar across phylogenetic groups. For instance, motif 1, motif 2, and motif 3 present in all the groups. For instance, motifs 1, 2, and 3 were present in all groups; however, group I contained motifs 1, 2, 3, 5, and 7, with exceptions for *BnaA.GeBP9/BnaC.GeBP25* (lacking motif 7) and *BnaA.GeBP1* (lacking motifs 5 and 7). Group II *BnaGeBPs* contained motif 1, motif 2. Motif 3, motif 4, motif 6, and motif 8 with exception for *BnaC.GeBP22, BnaC.GeBP23*, and *BnaC.GeBP24* lack motif 6 and motif 4, and *BnaC.GeBP21* and *BnaC.GeBP34* lack motif 4. The group III contained motif 1, motif 2, motif 3, motif 4, motif 6, motif 9, and motif 10 with exception for *BnaC.GeBP28* contained only motif 1, motif 2, and motif 3 (**Figure 2**). A comparative motif architecture analysis revealed that motifs 1, 2, and 3 exhibit significant sequence homology to the core DNA-binding domain of DUF573; motifs 1 and 4 contain a nuclear localization signal (NLS) sequence, and motifs 1, 2, and 4 contain non-typical leucine zipper regions (**Supplementary Figure 1**). These findings indicate the conserved characteristics of these motifs and their potential roles in DNA recognition, dimer stabilization, and nuclear trafficking.

**Figure 3:**
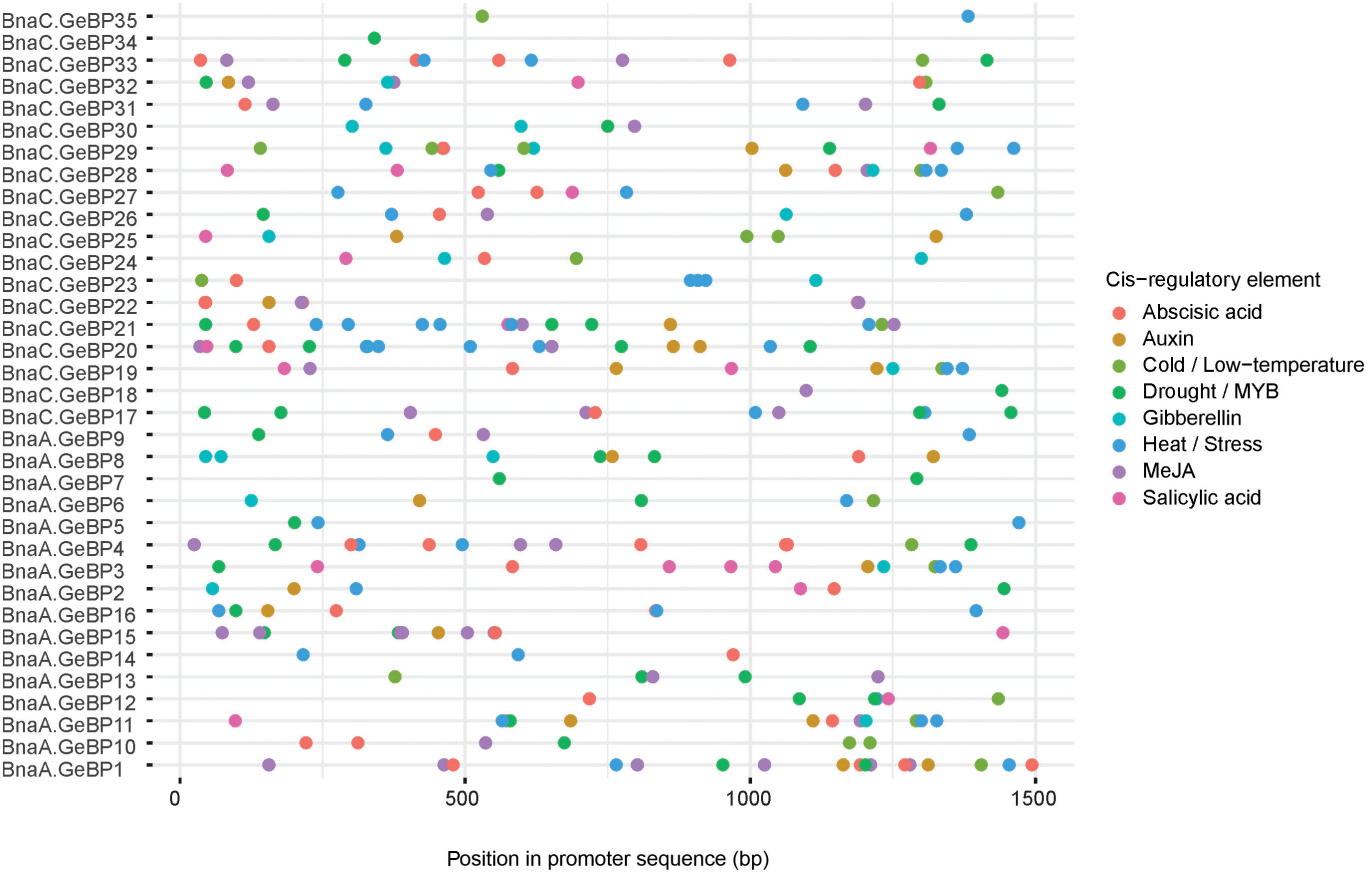
Distribution of hormone- and stress-responsive *cis*-regulatory elements in the promoter regions of *BnaGeBP* genes. The 1500 bp upstream promoter sequences of *BnaGeBP* genes were analyzed for *cis*-acting regulatory elements related to phytohormone signaling and abiotic stress responses. Different colored symbols represent various *cis*-elements associated with abscisic acid (ABA), auxin, gibberellin, methyl jasmonate (MeJA), salicylic acid, drought-responsive MYB binding sites (MBS), low-temperature-responsive elements (LTR), and other stress-related motifs. The x-axis indicates the position of *cis*-elements within promoter regions, while the y-axis represents individual *BnaGeBP* genes. The abundance and distribution of these elements suggest potential involvement of *BnaGeBP* genes in hormone-mediated signaling and environmental stress adaptation.

The promoter analysis of *BnaGeBP* genes revealed the presence of numerous hormone- and stress-responsive *cis*-regulatory elements, suggesting that these genes may participate in multiple signaling and environmental response pathways. Several phytohormone-responsive elements, including abscisic acid-responsive elements (ABRE), auxin-responsive elements (AuxRR-core and TGA-element), gibberellin-responsive elements (GARE-motif and P-box), methyl jasmonate-responsive elements (CGTCA-motif and TGACG-motif), and salicylic acid-responsive elements (TCA-element), were widely distributed across the promoter regions of the *BnaGeBP* genes. Among these, MeJA-responsive elements were particularly abundant, indicating a potential role of *BnaGeBP* genes in jasmonate-mediated stress and defense responses (**Figure 3**).

In addition to hormone-responsive elements, several abiotic stress-related cis-elements were identified. MYB binding sites (MBS), associated with drought inducibility, were detected in multiple promoters, suggesting involvement in drought stress responses. Low-temperature-responsive elements (LTR) were also identified in several genes, indicating possible functions under cold stress conditions. Furthermore, stress-responsive elements such as STRE and DRE-related motifs were present, implying potential roles in salt and general stress signaling pathways (**Figure 3**). Overall, the diverse distribution of hormone- and stress-associated cis-regulatory elements in the promoter regions suggests that *BnaGeBP* genes may function as important regulators involved in plant growth, hormone signaling, and adaptation to multiple environmental stresses.

### Chromosomal distribution and gene duplication analyses of the GeBP genes

**Figure 4:**
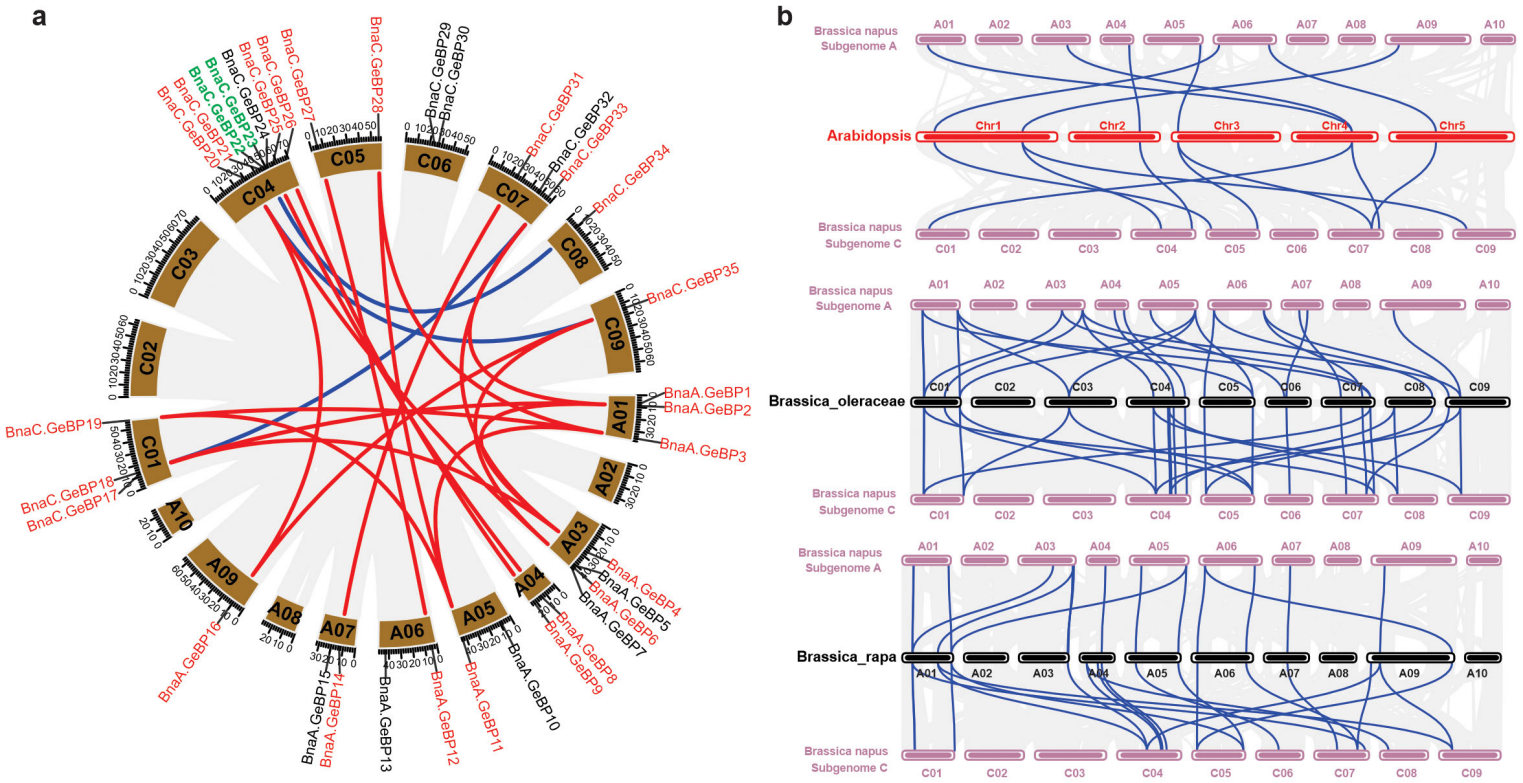
Chromosomal distribution, duplication events, and syntenic relationships of *BnaGeBP* genes. **(a)** Chromosomal distribution of *BnaGeBP* genes and Circos plot illustrating duplication events and predicted syntenic relationships within the *Brassica napus* genome. Black lines represent segmental duplication links between chromosomes. Genes highlighted in red and blue indicate segmentally duplicated gene pairs located in subgenome A and subgenome C, respectively. **(b)** Syntenic relationships of GeBP genes among *Brassica napus*, *Raphanus sativus*, *Brassica rapa*, and *Brassica oleracea*. Blue connecting lines represent conserved syntenic blocks and orthologous genomic regions between species.

The genomic positioning of *BnaGeBP* genes reveals an unequal distribution across all chromosomes. Specifically, chromosome C04 contains the highest number of *BnaGeBP* genes (7), followed by chromosome A03 (4 genes). Chromosomes A01, C01, and C07 each contain 3 *BnaGeBP* genes, while chromosomes A04, A05, A06, A07, C05, and C06 each harbor 2 *BnaGeBP* genes. Chromosomes A09, C08, and C09 contain a single *BnaGeBP* gene. Notably, none of the *BnaGeBP* genes are located on chromosomes A02, A8, A10, C02, or C03 (**Figure 4a**).

To explore *BnaGeBP* duplication events, we assessed segmental and tandem duplications among the *BnaGeBP* genes. This study identified 20 pairs of segmental duplication events and a single tandem duplication event involving 23 *BnaGeBP* genes. These duplication events were located on chromosomes A01, A03, A04, A05, A06, A07, A09, C01, C04, C05, C07, C8, and C09 (**Figure 3a**; **Supplementary Table 2**). Furthermore, selection pressure on duplicated *BnaGeBP* genes was assessed by calculating the ratios of nonsynonymous (Ka) to synonymous substitutions (Ks) for the paralogous gene pairs. The Ka/Ks ratios for these gene pairs ranged from 0.10 (*BnaA.GeBP11/BnaC.GeBP28*) to 0.64 (*BnaA.GeBP8/BnaC.GeBP25*), and the divergence times ranged from 1.69 Mya (*BnaA.GeBP9/BnaC.GeBP26*) to 22.33 Mya (*BnaC.GeBP21/BnaC.GeBP34*) (**Supplementary Table 2**). Additionally, the Ka/Ks ratios for all paralogous genes were less than 1, indicating purifying selection acting on *BnaGeBP* genes.

To elucidate the evolution of *BnaGeBP* genes, a comparative interspecific synteny analysis was performed. The interspecific collinearity analysis revealed a strong orthologous relationship among the *BnaGeBP* members; specifically, 14, 34, and 28 collinear pairs were identified between Arabidopsis, *B. rapa*, and *B. oleracea*, respectively (**Figure 4b**; **Supplementary Table 3**). Our study indicated structural, functional, and evolutionary convergence of the GeBP genes across diverse species.

### Spatial expression pattern of *B. napus* GeBP genes

**Figure 5:**
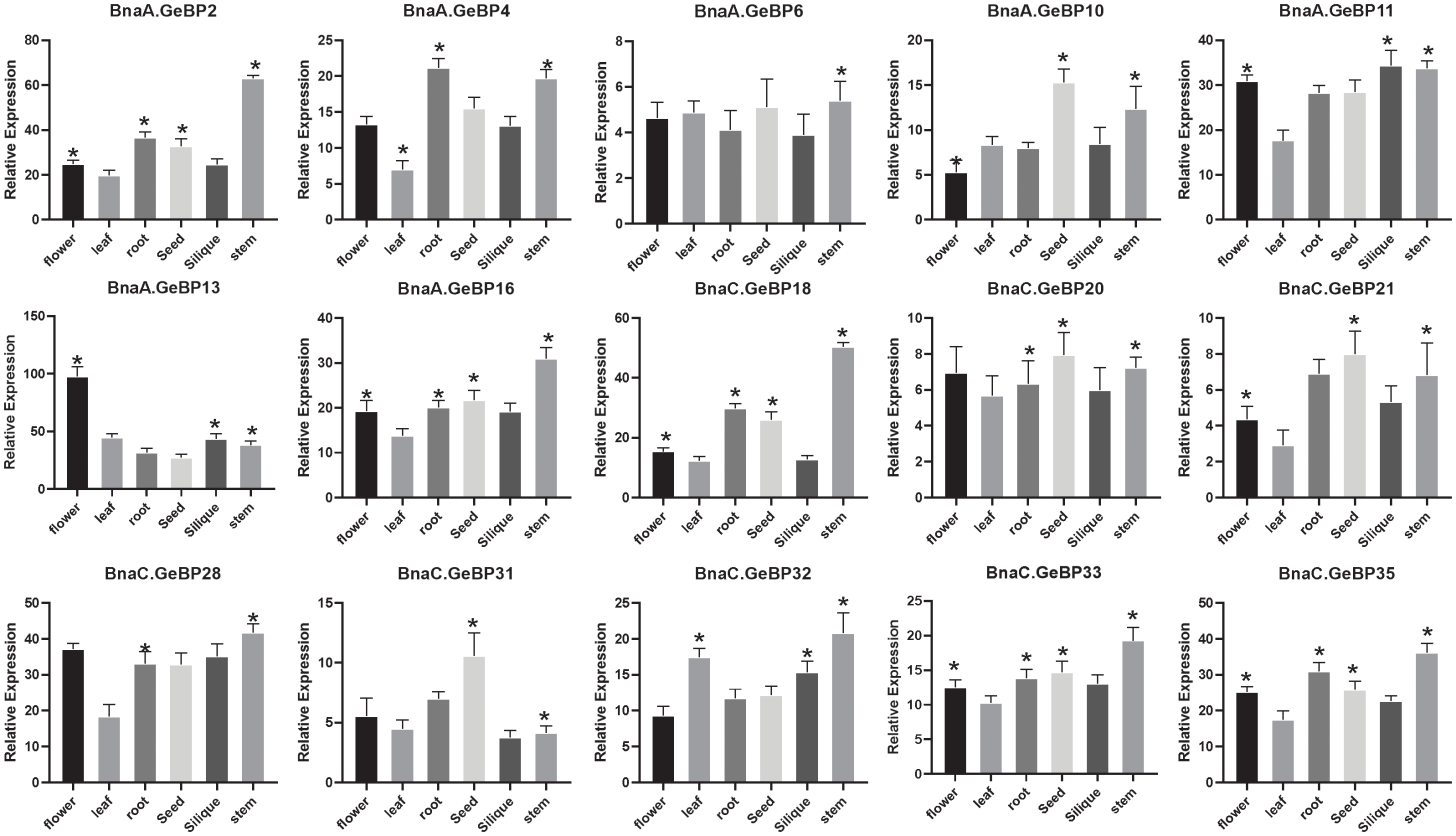
Spatial expression profiles of the *BnaGeBP* gene family in different tissues of *B. napus* by RT-qPCR. Error bars represent the standard deviation (SD) of the means from three replicates. Asterisks indicate statistically significant differences in expression (*P* < 0.05).

To gain preliminary insight into the potential functions of the *BnaGeBP* gene family in *B. napus*, tissue-specific expression patterns of 15 randomly selected *BnaGeBP* genes were analyzed using RT-qPCR. The results indicated that *BnaGeBP* genes exhibit tissue-specific expression patterns associated with development. For example, *BnaA.GeBP4, BnaA.GeBP6, BnaA.GeBP11, BnaA.GeBP16, BnaC.GeBP20, BnaC.GeBP21, BnaC.GeBP28, BnaC.GeBP32, BnaC.GeBP33*, and *BnaC.GeBP35* displayed moderate expression in all tissues examined. Notably, *BnaA.GeBP2* and *BnaC.GeBP18* exhibited high expression in stems, while *BnaA.GeBP13* was highly expressed in flowers and *BnaC.GeBP31* in seeds (**Figure 5**).

### Temporal expression of *B. napus* GeBP genes under salinity and drought stress

**Figure 6:**
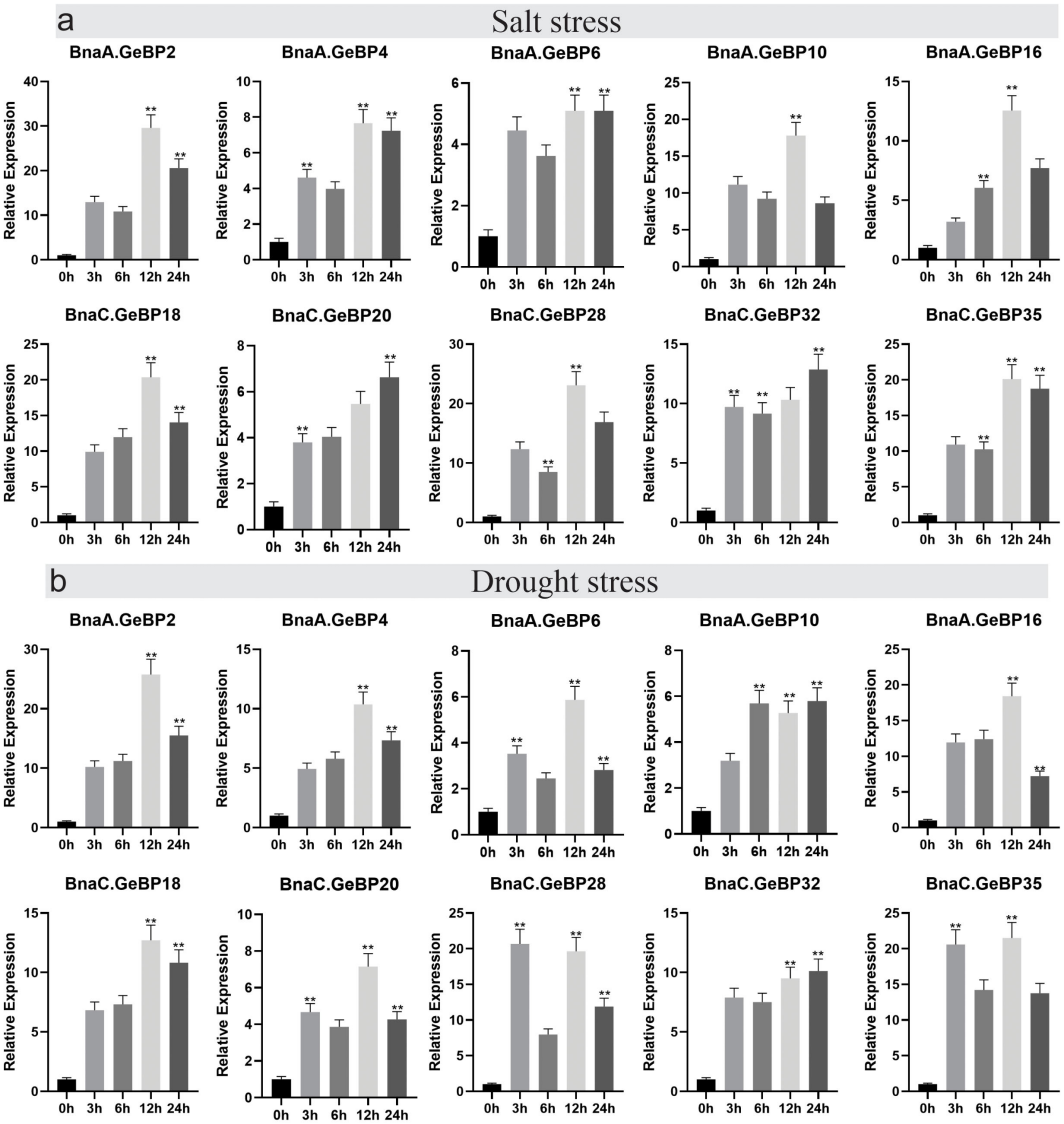
Temporal expression patterns of *BnaGeBP* under **(a)** salt and **(b)** drought stress in *B. napus* following intervals of 0 h, 3 h, 6 h, 12 h, and 24 h. The 0 h samples served as controls. Error bars represent SD of the means from three replicates. Asterisks indicate statistically significant differences in expression between the control and stress groups (*P* < 0.05).

To investigate the effects of salinity and drought stress on *BnaGeBP* expression, this study analyzed the relative expression of *BnaGeBP* in *B. napus* at various time intervals. The expression of selected *BnaGeBP* genes was significantly upregulated at 12 h following salt stress treatment. *BnaC.GeBP20* and *BnaC.GeBP32* exhibited significant upregulation at the 24 h time interval (**Figure 6a**; **Supplementary Table 5**). Several salt stress-responsive *BnaGeBP* genes, including *BnaA.GeBP2, BnaA.GeBP4*, and *BnaA.GeBP6*, contained STRE- and DRE-related cis-regulatory elements in their promoter regions, suggesting their potential involvement in salt stress signaling pathways (**Figure 3**).

Similarly, under drought stress (PEG6000), the majority of *BnaGeBP* genes were significantly upregulated at 12 h after exposure to PEG6000. *BnaC.GeBP28* and *BnaC.GeBP35* was significantly upregulated at 3h and 12h after drought stress while *BnaA.GeBP10* significantly peak at 6h and 24h. Lastly, *BnaC.GeBP32* expression upregulated temporally and peak at 24h (**Figure 6b**; **Supplementary Table 6**). Promoter analysis revealed that drought-responsive genes such as *BnaA.GeBP2, BnaA.GeBP4, BnaA.GeBP6, BnaA.GeBP10, BnaA.GeBP16, BnaC.GeBP18, BnaC.GeBP20, BnaC.GeBP28, BnaC.GeBP32*, and *BnaC.GeBP35* harbored MYB/MBS and MYC-related cis-elements associated with drought inducibility, supporting their stress-responsive expression patterns under drought treatment (**Figure 3**).

### Temporal expression of *B. napus* GeBP genes under temperature extremes

**Figure 7:**
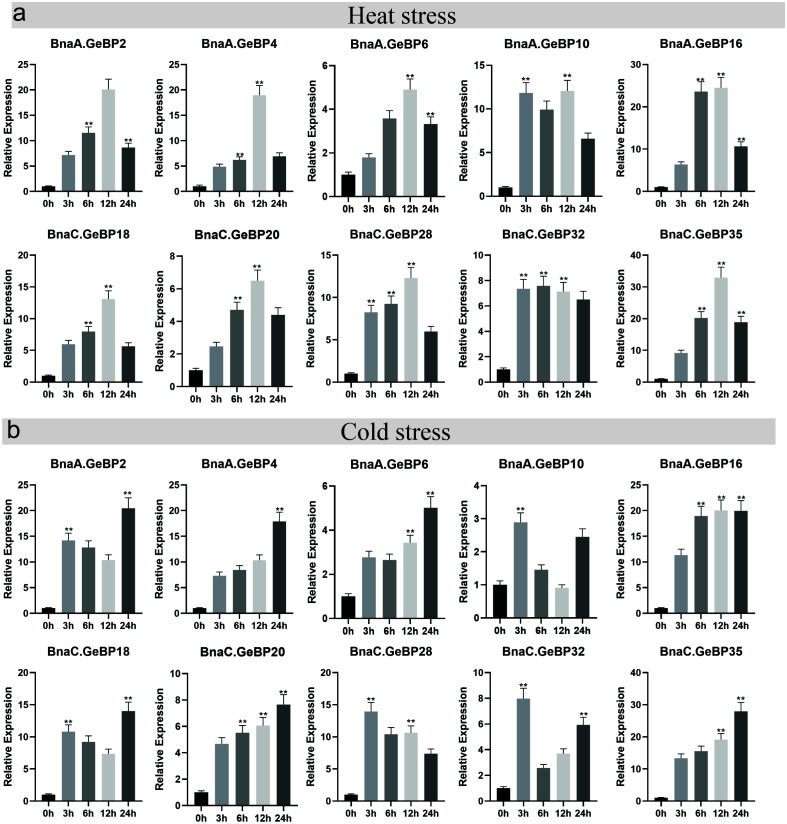
Temporal expression patterns of *BnaGeBP* under **(a)** heat (30 °C) and **(b)** cold (4 °C) stress in *B. napus* following intervals of 0 h, 3 h, 6 h, 12 h, and 24 h. The 0 h samples served as controls. Error bars represent SD of the means from three replicates. Asterisks indicate statistically significant differences in expression between the control and stress groups (*P* < 0.05).

We also investigated the expression of *BnaGeBP* genes at temperature extremes, including cold and heat. We observed that *BnaGeBP* gene expression increased temporally, peaking at 12h under heat stress for genes such as *BnaA.GeBP2, BnaA.GeBP6, BnaC.GeBP18, BnaC.GeBP20, BnaC.GeBP28*, and *BnaC.GeBP35*. *BnaA.GeBP16* was significantly upregulated at 6h and 12h, whereas BnaC.GeBP32 exhibited decreasing expression trends over time (**Figure 7a**; **Supplementary Table 7**). Several heat-responsive *BnaGeBP* genes, including *BnaA.GeBP2, BnaA.GeBP4, BnaA.GeBP6, BnaC.GeBP18, BnaC.GeBP20*, and *BnaC.GeBP35*, contained stress-associated cis-elements such as STRE and other general stress-responsive motifs, indicating that these promoter elements may contribute to the transcriptional activation of *BnaGeBP* genes during heat stress conditions.

For cold stress, the expression of *BnaA.*GeBP2 and *BnaC.GeBP15* peaked significantly upregulated at 24 h, while *BnaA.GeBP10* and *BnaC.GeBP32* showed significant high expression at 3 h after exposure to cold stress. However, for genes *BnaA.GeBP4, BnaA.GeBP6, BnaA.GeBP16, BnaC.GeBP20*, and *BnaC.GeBP35* ascending expression pattern with peak at 24h significantly. However, *BnaC.GeBP28* exhibited descending expression pattern temporally with minimum expression at 24h after cold treatment (**Figure 7b**; **Supplementary Table 8**). Cold-responsive genes, including *BnaA.GeBP2, BnaA.GeBP10*, and *BnaC.GeBP18*, contained low-temperature-responsive LTR elements in their promoter regions, suggesting their possible regulatory roles in cold stress adaptation.

### Subcellular location of GeBP genes

**Figure 8:**
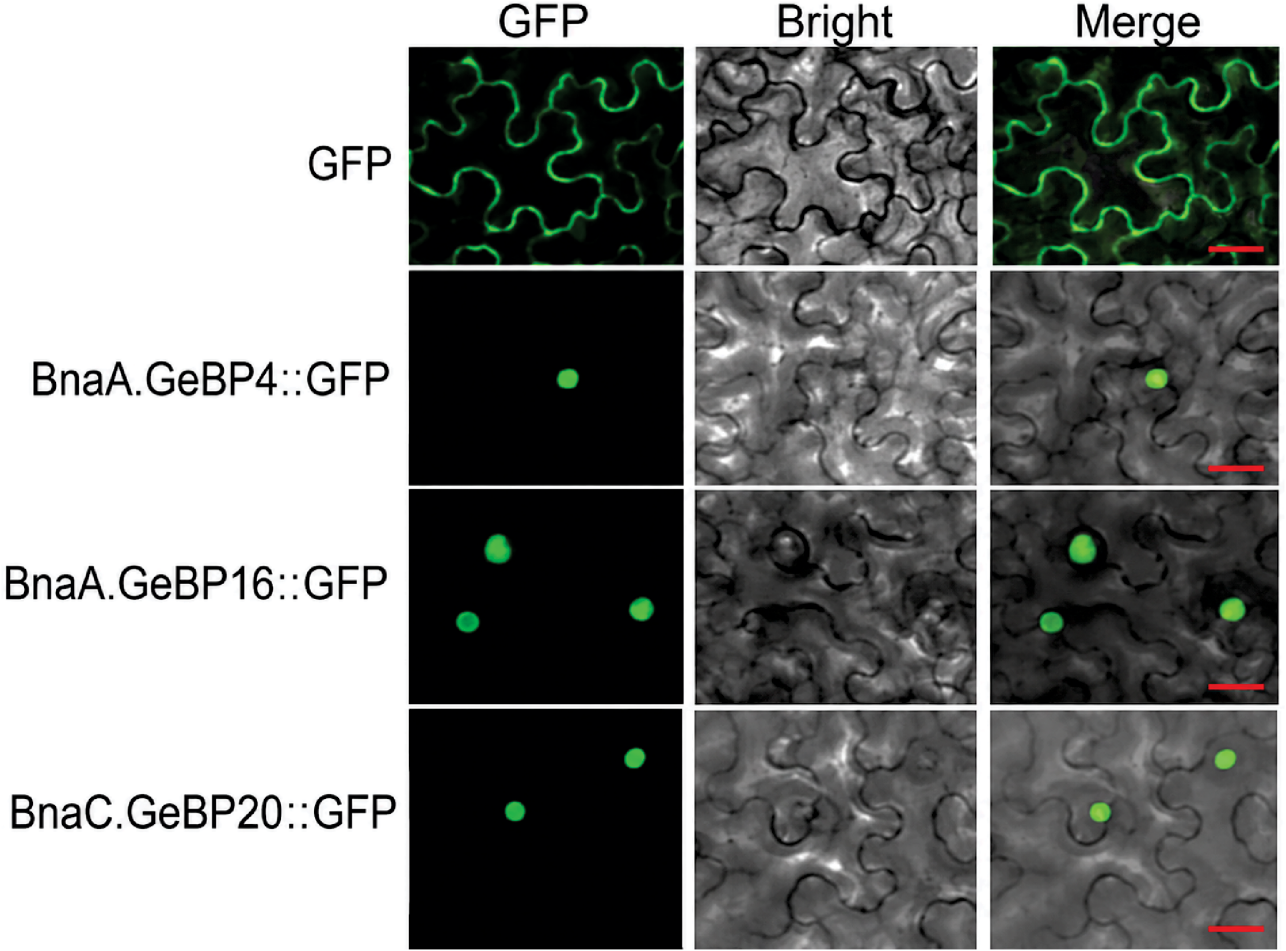
Transient expression of selected *BnaGeBP* genes, including *BnaA.GeBP4, BnaA.GeBP16*, and *BnaC.GeBP20*. Vector with only GFP is used as control. Bars = 50μm. GFP, green fluorescent protein.

*In-silico* subcellular localization prediction using WolfPsort indicated that BnaGeBP proteins are localized in various cellular compartments, including the nucleus, cytoplasm, and chloroplast (**Table 1**). To verify the reliability of the *in-silico* predictions, the transient expression of three randomly selected *BnaGeBP* genes, *BnaA.GeBP4, BnaA.GeBP16*, and *BnaC.GeBP20* was examined in tobacco leaves. The results demonstrated that the GFP signal of the fusion proteins was primarily localized in the nucleus for all selected *BnaGeBPs*. In contrast, the GFP signal in the control exhibited a broader distribution throughout the whole cell (**Figure 8**). These findings suggest that BnaGeBP proteins function within the nucleus.

## Discussion

As immobile organisms, plants are exposed to diverse environmental stresses, including drought, heat, cold, and salinity, which ultimately impact plant growth, development, and annual yield and productivity (Srivastava et al., 2026). In the context of climate change, the frequency and magnitude of these stresses are becoming increasingly intense and damaging, posing a challenge to global food security. To enhance crop resilience to both abiotic and biotic stresses, a comprehensive understanding of plant stress response and tolerance mechanisms is required. Plant have developed plenty of adoptive strategies, TFs offer one such strategies which regulate gene expression at transcriptional and posttranscriptional levels (Waseem et al., 2019; Wai et al., 2022; Waseem et al., 2022). Specifically, GeBP TFs, plant-specific domain-containing uncharacterized proteins 573 (DUF573) TFs, play critical roles in a range of biological processes, including plant development and resistance to environmental stresses (Huang et al., 2021; Cao et al., 2025). To date, GeBP TF have been identified in various plant species such as Arabidopsis (23 members) (Chevalier et al., 2007), apple (7 members) (Liu et al., 2023), *B. rapa* (20 members) (Wang et al., 2023), soybean (9 members), rice (13 members), tea (6 members), and tomatoes (11 members) (Cao et al., 2025). In the present study, 35 GeBP TF family members were identified in *B. napus* using bioinformatic approaches.

To predict the potential function of GeBP proteins in *B. napus*, an evolutionary and phylogenetic analysis of the GeBP protein with rice, tomato, maize, pepper, Arabidopsis, and soybean revealed that the entire GeBP gene family is clustered into three phylogenetic groups. Generally, members within the same subgroup may exhibit similar functions, which aids in understanding the potential biological functions of *BnaGeBP*. Cao et al. (2025) demonstrated that *SlGeBP* genes cluster into three groups, with group C exclusively containing GeBP members from monocot plants. Similarly, six pepper *CsGeBP* genes were clustered into group I and group II only (Zhou et al., 2023). In *B. napus*, *BnaGeBP* gene family members were clustered into three phylogenetic groups, with group I and group II containing GeBP members exclusively from dicot species. However, group III exhibited phylogeny specific to both monocot and dicot species (rice, maize, pepper, soybean, Arabidopsis, and tomato), indicating that the GeBP gene family evolved in multiple directions among these species.

Gene structure analysis, based on intron-exon distribution, indicated that *GmGeBP* genes contained no more than one intron (Liu et al., 2022). *SlGeBP* and *CsGeBP* genes were reported to have a maximum of one intron (Zhou et al., 2023; Cao et al., 2025). The investigation of gene structure feature revealed that *BnaGeBP* in *B. napus* ranged from zero to more than one intron. For instance, *BnaGeBP* genes in group II and III contained no intron while group I more than one intron. Furthermore, the analysis of conserved protein motifs showed that BnaGeBP proteins clustered on same phylogenetic clade shared similar motif compositions, which is aligned with previous findings in bamboo (Shan et al., 2020), pepper (Zhou et al., 2023), soybean (Liu et al., 2022) and tomatoes (Cao et al., 2025).

Gene duplication, alongside polyploidization, plays a critical role in the expansion and diversification of biological function and is considered a major driving force for the evolution of novelties (Ren et al., 2018; Shaheen et al., 2024). For instance, studies reported the presence wide range of segmental duplication events of GeBP in different species such as *Oryza rufipogon* (3 segmental duplication pairs), *O. sativa* ssp. *japonica* (3 segmental duplication pairs), *Zea mays* (7 segmental duplication pairs), *Brachypodium distachyon* (4 segmental duplication pairs), *O. sativa* ssp. *indica* (single segmental duplication pairs), *Sorghum bicolor* (4 segmental duplication pairs), *Setaria italica* (2 segmental duplication pairs), *Triticum aestivum* (5 segmental duplication pairs), cotton (7 segmental duplication pairs), and soybean (4 segmental duplication pairs) (Huang et al., 2021; Liu et al., 2022; Cao et al., 2025). In our study, we identified 20 pairs of segmental duplication events and a single pair of tandem duplication events. These results are consistent with previous studies and indicate that segmental duplication is a major driving force for the extension of GeBP genes in plants.

Activation of signal transduction networks also leads to the transcriptional regulation of plants. It follows the expression of stress-responsive genes that enhance the adaptive response of plants. In *B. rapa*, *BrGeBP5* and *BrGeBP17* exhibited higher expression levels under cold stress, while *BrGeBP3* and *BrGeBP14* showed increased expression under drought stress (Wang et al., 2023). Conversely, in *Solanum lycopersicum*, *SlGeBP3* and *SlGeBP4* demonstrated a higher transcriptional upregulation under drought stress, with *SlGeBP8* and *SlGeBP11* exhibiting the highest transcript levels under oxidative stress. The expression levels of *SlGeBP1*, *SlGeBP5*, and *SlGeBP9* were upregulated under both cold and dehydration stress, whereas *SlGeBP6* and *SlGeBP11* displayed antagonistic expression profiles (Cao et al., 2025). In Apple, The expression levels of *MdGeBP1, MdGeBP2, MdGeBP3, MdGeBP4, MdGeBP6* and *MdGeBP7* were significantly up-regulated to the highest values after 3 h of PEG treatment, while *MdGeBP5* was induced to be up-regulated at 1 h (Liu et al., 2023). In this study, several stress-responsive *BnaGeBP* genes contained one or more of these motifs, supporting the possibility that their transcriptional activation under salt, drought, heat, or cold stress is regulated through stress-specific promoter elements. For example, *BnaC.GeBP32* showed increased expression under salt and drought stress but reduced expression under cold and heat stress, suggesting that this gene may be preferentially involved in osmotic stress responses rather than temperature stress responses. This differential expression may result from the presence of promoter elements responsive to osmotic or ABA-mediated signaling, while cold- or heat-associated regulatory elements may be absent, less abundant, or functionally inactive in this promoter context. In contrast, *BnaA.GeBP20* and *BnaA.GeBP10* exhibited similar expression trends under salt and drought stress, respectively. *BnaC.GeBP28* was upregulated 12 hours after heat stress, with its expression level reaching a maximum at 24 hours under cold conditions. The consistency between promoter cis-regulatory element composition and stress-induced expression patterns further supports the potential involvement of *BnaGeBP* genes in abiotic stress signaling pathways. Genes harboring drought-responsive MYB/MBS motifs and low-temperature-responsive LTR elements generally showed enhanced expression under drought and cold treatments, respectively.

Although this study provides comprehensive insights into the evolutionary characteristics, promoter cis-regulatory elements, subcellular localization, and stress-responsive expression patterns of the *BnaGeBP* gene family, several limitations remain. The present work is primarily based on bioinformatic analyses and transcriptional profiling under abiotic stress conditions, and direct functional evidence for the biological roles of *BnaGeBP* genes is still lacking. Therefore, future studies should focus on functional validation using transgenic overexpression and gene knockout approaches, such as CRISPR/Cas9-mediated genome editing, to determine the precise roles of candidate *BnaGeBP* genes in stress tolerance. In addition, identification of downstream target genes, protein interaction partners, and signaling pathways will further clarify the molecular mechanisms underlying *BnaGeBP*-mediated stress responses in *B. napus*.

## Conclusion

The present study provides an in-depth, systematic analysis of the *BnaGeBP* gene family in *B. napus* using various bioinformatics approaches. It identified 35 *BnaGeBP* genes, which are unevenly distributed across all chromosomes. Whole-genome duplication analysis revealed 20 pairs of segmental duplications, with single tandem duplications indicating that segmental duplication played a major role in the expansion and evolution of *BnaGeBP* genes. Phylogenetic analysis of the identified *BnaGeBP* genes clustered them into three phylogenetic clades, alongside GeBP genes from several selected species. Exon-intron distribution and conserved motif sequence analysis revealed phylogenetic conservation within these groups, with a few exceptions. Tissue-specific expression patterns demonstrated that some *BnaGeBP* genes exhibited higher expression in specific plant parts, including flowers, seeds, and stems. RT-qPCR analysis of *BnaGeBP* genes under abiotic stresses–salinity, drought, heat, and cold–indicated their role in these applied stresses. Subcellular localization validation of selected genes confirmed their location within the nucleus. Briefly, this genome-scale study provides a foundation for further functional characterization of *BnaGeBP* genes concerning *B. napus* growth, development, and abiotic stress responses.

## Data Availability

The original contributions presented in the study are included in the article/**Supplementary Material**. Further inquiries can be directed to the corresponding authors.
