# Supplementary material for: Bioinformatic analysis, expression analysis, and subcellular localization of GeBP transcriptional regulator family in response to abiotic stress in Brassica napus
Source: Front Plant Sci. 2026 Jun 17;17:1858194. doi: 10.3389/fpls.2026.1858194 (PMC13318797; doi:10.3389/fpls.2026.1858194)
Supplement: Supplementary file 1 [file Supplementaryfile1.docx]

Supplementary data

Figures


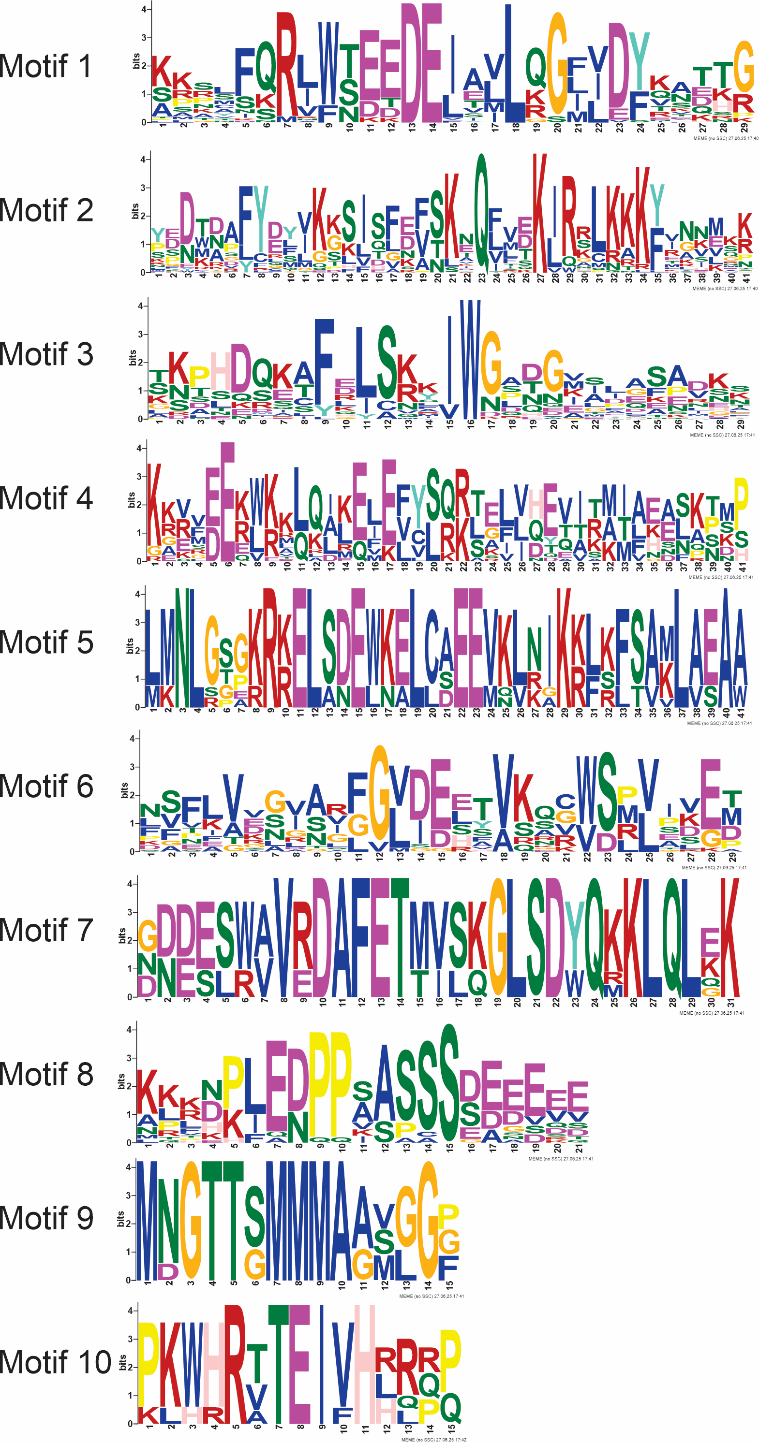


Figure 1. Sequence logos of conserved motifs predicted through MEME suite.
